# Prosthesis–Patient Mismatch and Aortic Root Enlargement: Indications, Techniques and Outcomes

**DOI:** 10.3390/jcdd10090373

**Published:** 2023-09-01

**Authors:** Ibrahim Talal Fazmin, Jason M. Ali

**Affiliations:** Royal Papworth Hospital, Cambridge CB2 0AY, UK; itf21@cam.ac.uk

**Keywords:** aortic root enlargement, prosthesis–patient mismatch, aortic stenosis, aortic valve surgery, adult cardiac surgery

## Abstract

Prosthesis–patient mismatch (PPM) is defined as implanting a prosthetic that is insufficiently sized for the patient receiving it. PPM leads to high residual transvalvular gradients post-aortic valve replacement and consequently results in left ventricular dysfunction, morbidity and mortality in both the short and long term. Younger patients and patients with poor preoperative left ventricular function are more vulnerable to increased mortality secondary to PPM. There is debate over the measurement of valvular effective orifice area (EOA) and variation exists in how manufacturers report the EOA. The most reliable technique is using in vivo echocardiographic measurements to create tables of predicted EOAs for different valve sizes. PPM can be prevented surgically in patients at risk through aortic root enlargement (ARE). Established techniques include the posterior enlargement through Nicks and Manouguian procedures, and aortico-ventriculoplasty with the Konno–Rastan procedure, which allows for a greater enlargement but carries increased surgical risk. A contemporary development is the Yang procedure, which uses a Y-shaped incision created through the non- and left-coronary cusp commissure, undermining the nadirs of the non- and left-coronary cusps. Early results are promising and demonstrate an ability to safely increase the aortic root by up to two to three sizes. Aortic root enlargement thus remains a valuable and safe tool in addressing PPM, and should be considered during surgical planning.

## 1. Introduction and Definition of Prosthesis–Patient Mismatch

Aortic valve replacement is one of the most performed procedures in adult cardiac surgical practice. Prosthesis–patient mismatch (PPM) was first described in 1978 as a complication of aortic valve replacement [1]. It is defined as occurring when a prosthetic valve effective orifice area (EOA) is too small relative to the size of the patient it is being implanted in. When indexed relative to a patient’s body surface area (BSA), it is expressed as the indexed EOA (iEOA). The degree of PPM can be expressed using the iEOA as follows: ≤0.85 cm^2^/m^2^—moderate PPM and ≤0.65 cm^2^/m^2^—severe PPM [2]. The definition has been recently updated by the valve academic research consortium 3 (VARC 3) criteria to also account for body mass index (BMI), as a high BSA would lead to the over-indexing of the iEOA and the overestimation of PPM. Thus, when BMI > 30 kg/m^2^, mild PPM is present; moderate PPM is present when iEOA < 0.70 cm^2^/m^2^ and severe PPM is present when iEOA is <0.55 cm^2^/m^2^ [3,4] (Table 1). The incidence of PPM shows variation across different studies and patient cohorts, but a recent meta-analysis demonstrated an incidence of 53.7% (with a range of 6.1–93.8% in included studies), highlighting that it is a common phenomenon [5]. It impacts the prostheses used in both surgical (SAVR) as well as transcatheter (TAVR) aortic valve replacement. Therefore, it is important for heart teams to understand PPM, its clinical impact, and strategies to account for and mitigate PPM, which include aortic root enlargement (ARE).

## 2. Measuring and Predicting PPM

The measurement of prosthesis–patient mismatch (PPM) holds significant importance in various stages of both surgical and transcatheter intervention, including preoperative, intraoperative, and postoperative phases. Preoperatively, the assessment of PPM serves to guide the selection of appropriate valve choices. It aids in determining the optimal valve size that would mitigate the occurrence of PPM in potentially high-risk patients, such as those with a high BMI or poor LVEF. In some cases, it might highlight the necessity of performing root enlargement. In Table 2 and Table 3, an example is highlighted using the Medtronic Hancock II and Carpentier–Edwards Perimount Magna prosthetic valve, simulating predicted iEOA for a variety of patient BSA values and valve sizes taken from the literature [6]. Using this table, we can see that a patient with a BSA of 2 m^2^ will require a minimum size 27 Hancock II or size 21 Perimount Magna valve to avoid PPM. Furthermore, intraoperative PPM measurements facilitate a comprehensive evaluation of the implanted valve, thereby predicting the likelihood of PPM in the specific patient. Therefore, PPM assessment serves as a vital component in risk stratification, enabling the identification of patients who require closer follow-up and monitoring.

There are several methods that can be used to predict the iEOA prior to intervention. These are evaluated in a study by Bleiziffer et al. [7] who compared four different methods and calculated the correlation with postoperative in vivo transthoracic echocardiographic (TTE) measurements:

- Method 1—Using in house TTE data obtained from patients 6 months postoperatively to create “home-grown” iEOA charts (r = 0.62);
- Method 2—Using the geometric orifice area based on static parameters (the valve internal diameter specified by the manufacturer) (r = 0.27);
- Method 3—Using commercial iEOA charts, which are produced by using data obtained through various methods but can include in vitro measurements (varies based on manufacturer; r = 0.27–0.59 for four different valve types);
- Method 4—Using published EOA data from the literature (r = 0.53).

The authors concluded that the best methods were methods 1 and 4. The most reliable sources of data to make preoperative predictions about iEOA are echocardiographic studies involving large numbers of patients for each valve size. In the absence of data in in the literature, for example, in new valves, “Method 1” of creating an institutional database of TTE-measured EOA values should be used. Furthermore, use of in vitro data should be cautioned against. This is supported by a recent meta-analysis [5], which observed that regardless of whether predicted (by manufacturer or published in vivo data) or measured iEOA is used, the same correlation with outcomes (perioperative mortality) is found. Furthermore, each different method of preoperatively predicting iEOA showed different degrees of statistical heterogeneity when compared across the studies included in the meta-analysis, with Doppler echocardiography being the most reliable.

Counterarguments, however, have emerged. Two studies by Ternacle et al. showed that TTE-derived iEOA values demonstrate high variability, and suggest that this method overestimates PPM, compared with using predicted iEOA derived from manufacturer reference values [8,9]. Furthermore, they found that predicted iEOA correlated better with hemodynamic parameters (trans-prosthetic gradients and high residual gradients), whilst neither predicted nor measured iEOA correlated with clinical outcomes. The authors suggest that drawbacks of TTE measurements include inter-operator variability and the susceptibility of TTE to underestimate EOA in low-flow states [8,9]. However, a major drawback in the generalisability of these studies is that they looked at TAVR prosthetics as opposed to SAVR.

Another argument highlighting the limitations of TTE measurements is put forward by Vriesendorp et al. [10], who analyzed the predictive value of iEOA charts in a homogenous cohort of patients. They constructed “train” and “test” subgroups, wherein they measured EOA in the “train” cohort and tested the predictive value by comparing with the post-implant EOA in the “test” cohort. They demonstrated a large variation in measured EOA for each size of valve and a high degree of misclassification of PPM in the test cohort. Nonetheless, even using in vitro measurements, the correlation between projected iEOA (derived from iEOA charts created using TTE data from the train subset) and measured iEOA (in the test subset) was poor (r = 0.50) [10].

## 3. Clinical Impact of PPM

The impact of PPM on patients is profound. It can lead to higher morbidity and mortality and persistent symptoms, and accelerates the degeneration of bioprosthetic valves [2,11,12]. The most recently published large-scale clinical study involved 16,423 patients and demonstrated that severe PPM (adjudicated based on published EOA data and VARC3 criteria) impacted long-term (10 year) mortality, as well as leading to increased readmissions with heart failure [13]. However, the same study suggested that moderate PPM had a negligible effect. Therefore, some groups suggest that we may be too aggressive in taking steps to avoid moderate PPM, conducting more extensive surgeries for limited prognostic benefit [4].

There have been five meta-analyses looking at patient outcomes related to PPM. Sa et al. [5] included 108,182 patients with moderate and severe PPM, and demonstrated increased peri-operative mortality, and also mortality 1, 5, and 10 years after surgery. In a subgroup analysis, the mortality impact was worse in the severe PPM group compared to the moderate PPM group. Dayan et al. [14] assessed 40,381 patients (of which 813 had TAVR), and showed perioperative mortality was 56% higher and overall mortality 26% higher in the PPM group. PPM also has an increased effect of worsening mortality in patients aged <70 or with concomitant coronary artery bypass grafting (CABG). When divided into subsets of severe and moderate PPM, severe PPM caused increased mortality in the perioperative period and in the long-term, but moderate PPM only did so in the perioperative period, indicating perhaps a vulnerability of the myocardium to increased afterload immediately post-surgery. Chen et al. [15] included 14,874 patients and showed that PPM increased mid-term (5-year) and long-term (10-year) mortalities by 42% and 52%, respectively. They showed that this was the case for all patient sub-populations with severe PPM, but not those with moderate PPM. Younger patients, women, and patients with poor preoperative left-ventricular ejection fraction (LVEF) showed worse long-term outcomes in the presence of any degree of PPM. In patients with impaired LVEF, both moderate and severe PPM increased both perioperative and long-term mortality. Takagi et al. [16] included 16,021 patients and demonstrated a 31% increased risk of late mortality in PPM. However, when stratifying patients according to severe or moderate PPM, only those with severe PPM showed an increase in hazard ratio for mortality. Head et al. [17] included 27,186 patients and demonstrated a 34% increase in all-cause, long term mortality for all definitions of PPM. Additionally, when stratified by degree of PPM, both moderate and severe PPM were associated with increased all-cause and cardiac mortality. Therefore, PPM is associated with increased morbidity and mortality, with severe PPM being strongly associated with worse outcomes, although the impact of moderate PPM is not consistent across the published literature.

A particular subgroup of patients who are at significant risk of poor outcomes due to PPM are those with poor LVEF. Blais et al. [18] showed that in patients with LVEF ≥ 40%, early mortality was relatively low with non-severe or moderate PPM (mortality rate 2–5%). However, for patients with LVEF < 40%, mortality was 16%, and it was 77% for both those with moderate and those with severe PPM. The authors suggest that this is because an impaired LV is more vulnerable to increased afterload. This will have implications for the management of potential PPM when deciding to treat these patients, as a poor LVEF will be a strong indication to undertake strategies to mitigate potential PPM.

## 4. Management of PPM by Aortic Root Enlargement

Given the consequences of PPM, efforts to address it must be considered—as per the 2021 ESC/EACTS Guidelines for the management of valvular heart disease, “*Efforts to prevent PPM should receive more emphasis to improve long-term survival after either SAVR or TAVI*” [19]. A proposed management scheme for PPM is described in a review by Bilkhu et al. [20], as shown below.

Preoperatively predict the iEOA of a chosen prosthesis for a patient, and then:

1. Proceed with the selected prosthesis if the predicted iEOA is >0.85.
2. If the predicted iEOA is ≤0.85 then:
  - Accept PPM in certain clinical contexts;
  - Choose a prosthetic with larger EOA;
  - Carry out an aortic root enlargement.

In terms of choosing a prosthetic with a larger EOA, some valve designs have larger EOA values for the same size of valve. This is demonstrated in Table 2 and Table 3 above, which show that for the same label size, a Carpentier–Edwards Perimount Magna has a larger EOA than a Medtronic Hancock II prosthesis. Additionally, newer-generation prosthetic valves are being produced, which have larger EOAs with the same external diameter. Stentless valves such as the Corcym Perceval valves also have larger EOAs [21], as do TAVR prostheses. Sutureless aortic valves [22] and TAVR valves [23] both offer larger EOAs due to the lack of a sewing ring [24]. Certainly, TAVR appears to confer advantages over SAVR in terms of a reduced incidence of PPM. Studies show that in TAVR, the incidence of moderate PPM is around 6–46%, and for severe PPM the incidence ranges from 0 to 15% [25]. A meta-analysis demonstrated a 77% relative risk reduction in TAVR patients of developing PPM [26], and a recent analysis of the PARTNER 2 trial data showed that there was a decreased incidence of PPM in the TAVR group (9.3%) vs. the SAVR group (27.9%) [9]. TAVR valves were advantageous in terms of having reduced transvalvular gradients, greater EOAs and reduced risk of PPM compared to SAVR [25]. However, newer-generation TAVR devices seem to have diminished advantages (i.e., higher rates of PPM) due to a reduction in EOA on account of external skirts designed to mitigate paravalvular leak [27]. Therefore, a variety of patient- and operation-related factors must be considered on a case-by-case basis and after undertaking a heart team multidisciplinary discussion before counselling patients and obtaining informed consent to proceed with an aortic root enlargement.

Indications for aortic root enlargement are risk of postoperative PPM, as defined above. Severe PPM would be a strong indication, with moderate PPM being a more controversial indication due to the conflicting evidence relating to its impact on clinical outcomes, with some studies suggesting a negligible effect of moderate PPM on morbidity and mortality [13]. Certainly, some authors urge caution with being too aggressive in prospectively avoiding PPM, as the operative risk of a root enlargement will outweigh the benefit in treating a moderate PPM [4]. Some patient-related factors will also influence this decision. For example, patients with a poor LVEF will be at high risk of early mortality if there is PPM post-AVR [18]. Demographic factors are also important. Sa et al. discuss this from a global health perspective in their recent meta-analysis [5]: in developed countries, a common scenario is deciding to implant a small, 21 mm prosthesis in a short, obese, frail elderly female patient with calcific aortic stenosis and a low functional baseline. A degree of PPM would be acceptable in this case [5]. In a developing country, a possible scenario would be having to replace rheumatic aortic valves in younger, more active patients wherein avoiding PPM would be a higher priority [5]. A further dimension to consider is that patients in developed countries will be implanted with newer prosthetics with better hemodynamic profiles [5]. Finally, the choice of a mechanical versus bioprosthetic valve should also be considered. Although calcific aortic stenosis predominantly affects older patients, younger patients with diseases such as bicuspid aortic valves are suitable candidates for mechanical valve prostheses, which, due to their construction, tend to have larger EOAs and better hemodynamic performance compared to similarly sized bioprosthetic valves [6,28].

## 5. Established Techniques of Aortic Root Enlargement

**Figure 1:**
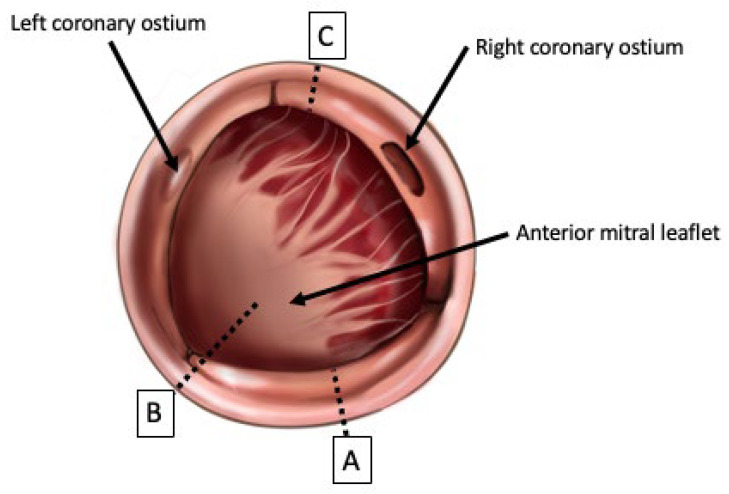
Surgical anatomy of the aortic root, with valve leaflets excised, with incision lines for the Nicks procedure ((**A**): through the non-coronary cuspJ); the Manouguian procedure ((**B**): through the commissure between the non- and left-coronary cusps and extending into the anterior mitral leaflet), and the Konno–Rastan procedure ((**C**): through the right coronary cusp and (not drawn) continuing into the interventricular septum via the aortic annulus). Informed by [29,31,36,37,38].

Aortic root enlargement techniques have traditionally been considered in two categories: posterior enlargement techniques (the Nicks and Manouguian techniques, Figure 1) and complex, less-commonly performed, anterior enlargement with aorto-ventriculoplasty (the Konno–Rastan procedure, Figure 1) [29,30,31]. ARE accounts for less than 1 in 10 of all aortic valve replacement procedures in the STS database [32] and for around 3% of all aortic valve surgeries in a recent meta-analysis of 213,134 patients [33], although it is increasingly being advocated for as PPM is being increasingly recognised as an Important issue to address [5,33,34,35].

### 5.1. Techniques of Aortic Root Enlargement

The Nicks procedure was first described in 1970 [36]. Briefly, it involves extending the aortotomy incision into the non-coronary sinus of the aortic root and then performing a patch repair to the defect to repair and enlarge the annulus. The Manouguian technique was first described in 1979 [37], and involves the same steps, but instead of cutting through the non-coronary sinus, the aortotomy incision is continued through the commissure between the left- and non-coronary sinuses, and may be extended to the anterior mitral curtain to allow for further up-sizing [29]. A variation of this technique is the Manouguian–Nunez procedure, whereby the incision stops short of being extended into the anterior mitral leaflet [31]. Additionally, the Manouguian technique involves opening the roof of the left atrium. Consequently, there is a degree of risk of mitral regurgitation with the Manouguian procedure [39].

The Konno–Rastan procedure, first described in 1975 [40], is a more complex operation and involves an anterior incision and aortoventriculoplasty. The aorta is transected anteriorly in a longitudinal fashion, and the cardiomyotomy carried through the right coronary sinus and aortic annulus and through to the interventricular septum. The anterior surface of the right ventricle is also opened, and the right ventricular outflow tract enlarged. A double-patch repair is then used to repair the defects in the aorta, interventricular septum and RVOT [38]. Care must be taken during this procedure to avoid injuring important structures: the initial incision through the right coronary cusp must pass near the commissure between the right and left coronary sinuses to avoid damaging the cardiac conduction system, and when incising the right ventricular free wall, care must be taken to avoid damaging the right coronary artery [31,38]. There is an additional risk of the formation of intracardiac fistulae between the chambers [31].

Although it carries an increased operative risk due to the complexity of the enlargement and repair, the Konno–Rastan procedure allows for more enlargement than the Nicks and Manouguian techniques. The Nicks procedure generally allows for enlargement by one size, the Manouguian by two sizes [31]; the Konno–Rastan procedure allows for greater enlargement than either, with up to three to four sizes of increase [41]. However, the Nicks procedure remains the most commonly performed procedure, perhaps due to the ease of carrying it out and the short learning curve suggested by some authors [31,42].

### 5.2. Outcomes of Aortic Root Enlargement Procedures

The “established” (i.e., Nicks and Manouguian) procedures for ARE have been extensively studied and demonstrated to be safe in experienced centers, although there are risks that much be considered at the time of surgical planning. Yu et al. conducted a meta-analysis, which included 8561 patients of whom 2570 underwent ARE [43]. The group, which underwent ARE, had increased cross-clamp and bypass times [43], and this is consistent with a large, contemporaneously published propensity-matched retrospective cohort study, which showed increased cross-clamp, bypass and total operative times [44]. Therefore, the additional operative steps required to conduct ARE prolong the operation, and risks of perioperative complications must be considered.

Studies have also examined the postoperative complications of ARE. There appears to be no increased risk of conduction defects (complete heart block, permanent pacemaker implantation), ischemic events (myocardial infarction, stroke), or re-operation for bleeding [35,43,44]. However, one study [44] did report an increased occurrence of respiratory failure in the ARE group (18.3% vs. 9.5%). In this study, ARE was not independently correlated with risk of increased mortality; however, postoperative respiratory failure was (HR 2.84) [44]. Hence, despite the association, postoperative respiratory failure ARE can nevertheless be performed safely.

ARE is effective in treating PPM, ”nd r’duces moderate and severe PPM while significantly increasing the postoperative aortic valve EOA [34,43,45]. The effect on perioperative mortality, however, is more contentious. Some studies report no increase in perioperative mortality [43,44]. Other studies report increased short-term mortality [34,35,46]. However, in two of these studies, when adjusting for confounders by matching cohorts or when excluding ARE performed concomitantly with other surgeries such as mitral valve surgery and coronary artery bypass grafting, the perioperative mortality risk was deemed not significant [34,35]. When considering long-term outcomes, Yu et al. [43] had a mean follow up of 7.8 years, and Sa et al. [33] had a median follow up of 3 years, while both show no difference in late mortality. Mehaffey et al. conducted a recent, large study including 5412 patients who underwent ARE from the Society of Thoracic Surgeons (STS) database, and demonstrated that although there was a short-term morbidity and mortality risk associated with ARE, the survival curves crossed over at the 3-year mark in favor of the ARE arm compared to 183,856 patients who underwent SAVR ± CABG without ARE [47]. Therefore, when considering the option of ARE, the short-term risk of increased complications and mortality must be weighed against the long-term advantage of addressing PPM in select patients. Decision-making will benefit from future randomized clinical trials, as all the evidence so far is derived from non-randomized studies.

## 6. Contemporary Developments in ARE: The Yang Procedure

**Figure 2:**
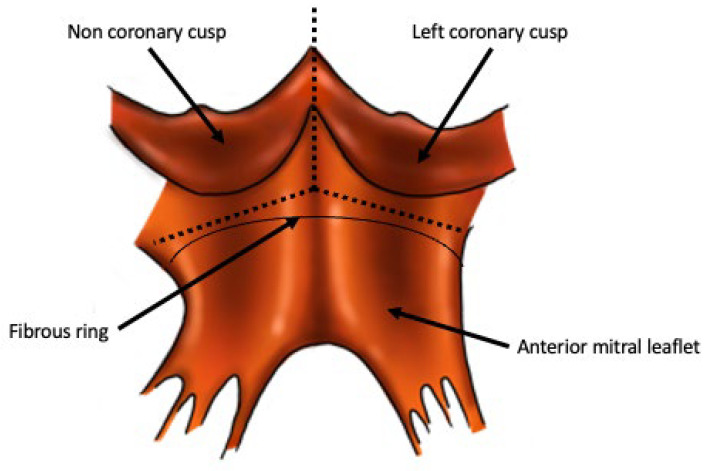
Incisions for the Yang procedure. The view of the aortic root is from the inside out, with excised aortic valve leaflets. The Y-shaped incision is created by incising between the non- and left-coronary cusps and extending the incision to undermine either cusp. Informed by [48].

The Y incision technique is a novel technique of aortic root enlargement that was first described in 2021 [48], and was subsequently named the “Yang procedure” [49]. It involves making an incision through the commissure between the non- and left-coronary cusps, into the aortomitral curtain, and extending it to create an inverted “Y” shape undermining the left and noncoronary cusps (Figure 2). A rectangular patch repair of the resultant defect is then carried out. The Yang procedure enlarges the fibrous part of the aortic root and thereby the annulus—similar to the Nicks and Manouguian techniques [48]. However, the Yang procedure does not enlarge the basal ring of the aortic root, in contrast to what is achieved in the Nicks, Manouguian and Konno–Rastan procedures [50].

A subsequent addition to this procedure was described in 2022, dubbed the “Roof technique”; this involves an incision and triangular-shaped patch repair of the ascending aorta above the previously described rectangular patch repair. This is undertaken via aortotomy closure, which allows for concomitant aortic enlargement in a proximal–distal fashion [51]. It enables the straightening out of any kinks between the native ascending aorta and the newly enlarged root, effacing the sinotubular junction and thus preparing the patient for a future valve-in-valve TAVR [50].

There are advantages to the Yang technique over the Nicks and Manouguian. Some surgeons may be hesitant about performing a Manouguian procedure due to the risk of mitral regurgitation, therefore stopping the incision before the anterior mitral leaflet (sometimes called a modified Manouguian or a Manouguian–Nunez procedure) [52]. This will protect the mitral valve, but will not allow for a significant up-sizing. The Yang procedure manages to create a significant upsizing while reducing risk to the mitral valve by extending the incision in an inverted Y-shape and undermining the left- and non-coronary cusps. This leaves a margin of tissue above the aortomitral curtain, reducing traction on the anterior mitral leaflet. Ultimately, the left atrium and mitral valve are not violated, and a greater degree of annular upsizing can be achieved [48]. In addition to ameliorating PPM, this will also allow for “future proofing” as the larger prosthetic will make a subsequent valve-in-valve TAVR easier to achieve [53].

There are concerns that a situation similar to subvalvular stenosis would arise as a result of the Yang procedure due to the LVOT and basal ring of the aortic root not being enlarged and the prosthesis being implanted in a supra-annular location [54]. However, the internal diameter of prosthetic valves is often smaller than the labeled diameter, due to structures such as the sewing ring and struts of the prosthesis and aortic annular tissue from the residual annulus [50]. The Yang procedure also allows for both the supra-annular and the intra-annular implantation of prosthetic valves [50]. Furthermore, not enlarging the basal ring will not have a functional consequence, since the basal ring is normally sized even with stenotic aortic valves, and is not the site of flow limitation [50]. This is supported by computed tomography imaging, which shows the implanted valve sitting on the LVOT like a “crown on a head”, with satisfactory hemodynamics, further supported by the mean pressure gradient across the LVOT remaining unchanged from preoperative measurements [50].

Additionally, there are concerns regarding the distortion of the aortic root anatomy, arising due to the significant upsizing achieved by this procedure. Firstly, over-sized prostheses being implanted could increase risks of compromising the closure of the aortotomy or causing coronary ostial obstruction [54]. Secondly, the rectangular patch repair of the aortic annulus causes a rotation of the left coronary ostium, which may increase the risk of coronary obstruction due to distortion and kinking [52,55]. The left coronary ostium will rotate by approximately 90°, and caution must be exercised if there is coronary artery disease, as not properly mobilizing the ostium before rotation could lead to luminal compromise [55]. Further investigations should be undertaken, such as radiological evaluations of the anatomical implications of manipulating the aortic root and left coronary ostium in this technique [55]. Finally, due to the tilting of the prosthetic valve during implantation, turbulent periprosthetic blood flow could lead to worsening bioprosthetic degradation [24]. However, this has been addressed with the addition of the “roof procedure”, which straightens out the sinotubular junction and ascending aorta, and postoperative-computed tomography shows a well aligned valve laying perpendicular to direction of blood flow [50].

The index case report [48] showed an increase of two valve sizes, with subsequent case reports showing increases of three [56], four [57] and five valve sizes [58], as the authors increased in experience with the procedure. Early outcomes of 50 consecutive patients undergoing the Yang technique have been reported. Median annular enlargement was by three valve sizes, and a reduction in mean aortic valve gradient from 40 mmHg to 7 mmHg from pre- to post-operative stages, respectively [59]. There was no operative mortality and no major complications (such as reopening for bleeding, chronic dialysis-dependent renal failure) except for one case of stroke, and there was zero mortality at 18 months follow-up [59]. However, only 25 of 50 the patients in this early outcome paper underwent the “roof technique”, although there was no significant hemodynamic difference in those the “roof technique” was carried out in compared to those where it was not [50]. Thus, the Yang procedure is a promising new technique, although data on long-term outcomes will not be available before widespread adoption can take place.

## 7. Conclusions

PPM is increasingly recognised as a significant complication/consequence of AVR. The role of moderate PPM is unclear, but severe PPM is strongly associated with poor long-term morbidity and mortality. Strategies to address PPM include sutureless prosthetics, TAVR and the usage of new-generation valves with bigger EOAs. Aortic root enlargement is another strategy to up-size aortic roots with two well-established procedures, the Nicks and Manouguian procedures, although these are rarely performed, and data on outcomes are limited to non-randomized studies. The Yang procedure is a recent development that allows for a much larger degree of enlargement, but there are no data on long-term outcomes. The significant increase in annulus size will not only help address PPM, but will also facilitate future valve-in-valve TAVR, providing a long-term solution for younger patients.

## Figures and Tables

**Table 1:** Definitions of PPM based on iEOA. Adjustments according to BMI are also included. PPM—prosthesis–patient mismatch, iEOA—indexed effective orifice area, BMI—body mass index.

Definition	iEOA	BMI Adjustment (>30 kg/m^2^)
Moderate PPM	≤0.85 cm^2^/m^2^	<0.70 cm^2^/m^2^
Severe PPM	≤0.65 cm^2^/m^2^	<0.55 cm^2^/m^2^

**Table 2:** Simulation of predicted iEOA for a range of patient BSA values and Medtronic Hancock II valve sizes. Moderate PPM values are highlighted in yellow, severe in red. BSA—body surface area, EOA—effective orifice area, PPM—prosthesis–patient mismatch.

Valve Size (mm)	EOA (cm^2^)	Indexed EOA (cm^2^/m^2^)			
21	1.20	0.80	0.69	0.60	0.53
23	1.40	0.93	0.80	0.70	0.62
25	1.60	1.07	0.91	0.80	0.71
27	1.80	1.20	1.03	0.90	0.80
29	2.00	1.33	1.14	1.00	0.89
		**1.50**	**1.75**	**2.00**	**2.25**
		**Patient BSA** (**m^2^**)			

**Table 3:** Simulation of predicted iEOA for a range of patient BSA values and Carpentier–Edwards Perimount Magna valve sizes. Moderate PPM values are highlighted in yellow. BSA—body surface area, EOA—effective orifice area, PPM—prosthesis–patient mismatch.

Valve Size (mm)	EOA (cm^2^)	Indexed EOA (cm^2^/m^2^)			
19	1.58	1.05	0.90	0.79	0.70
21	1.90	1.27	1.09	0.95	0.84
23	2.07	1.38	1.18	1.04	0.92
25	2.33	1.55	1.33	1.17	1.04
27	2.38	1.59	1.36	1.19	1.06
29	2.84	1.89	1.62	1.42	1.26
		**1.50**	**1.75**	**2.00**	**2.25**
		**Patient BSA** (**m^2^**)			

## Data Availability

Not applicable.
